# Biological Basis for Increased Sensitivity to Radiation Therapy in HPV-Positive Head and Neck Cancers

**DOI:** 10.1155/2014/696028

**Published:** 2014-04-03

**Authors:** V. Bol, V. Grégoire

**Affiliations:** Center for Molecular Imaging, Radiotherapy and Oncology, Institut de Recherche Expérimentale et Clinique (IREC), Université Catholique de Louvain (UCL), B1.5407 Avenue Hippocrate, No. 54-55, 1200 Brussels, Belgium

## Abstract

Although development of head and neck squamous cell carcinomas (HNSCCs) is commonly linked to the consumption of tobacco and alcohol, a link between human papillomavirus (HPV) infection and a subgroup of head and neck cancers has been established. These HPV-positive tumors represent a distinct biological entity with overexpression of viral oncoproteins E6 and E7. It has been shown in several clinical studies that HPV-positive HNSCCs have a more favorable outcome and greater response to radiotherapy. The reason for improved prognosis of HPV-related HNSCC remains speculative, but it could be owned to multiple factors. One hypothesis is that HPV-positive cells are intrinsically more sensitive to standard therapies and thus respond better to treatment. Another possibility is that HPV-positive tumors uniquely express viral proteins that induce an immune response during therapy that helps clear tumors and prevents recurrence. Here, we will review current evidence for the biological basis of increased radiosensitivity in HPV-positive HNSCC.

## 1. Introduction

Head and neck squamous cell carcinoma (HNSCC) is the sixth most common cancer worldwide with an annual incidence of approximately 400.000 cases. Although tobacco and alcohol consumption are the main risk factors for development of HNSCC, a causal link between Human papillomavirus (HPV) infection and a subgroup of head and neck cancers has been established, mostly in the oropharynx [1–4]. Incidence of HPV-positive oropharyngeal carcinomas (OPC) varies worldwide from approximately 25% to 80% and incidence is predicted to increase in the following years [5]. Among approximately 15 high-risk oncogenic HPV types that have been identified in the past years, HPV-16 is the most common type found in 87 to 90% of HPV-positive oropharyngeal cancers [6, 7].

Recent studies indicate that the expression of HPV-associated p16 (hereafter referred to as HPV/p16-positivity) in HNSCC is correlated with a better prognosis and improved response to conventional radiotherapy [8–12]. While HPV/p16 positivity seems to be associated with lower exposure to tobacco and alcohol and with younger age at the time of diagnosis, evidence is accumulating that HPV/p16-positive HNSCCs represent a separate clinical subgroup and that biological differences between these subtypes might have an impact on prognosis [13]. Here, we will review current evidence for the biological basis of increased radiosensitivity in HPV/p16-positive HNSCC.

## 2. The Role of HPV Oncoproteins E6 and E7 in Carcinogenesis

Human papillomaviruses comprise a large group that has been subdivided in low-risk and high-risk viruses, the last ones being associated with cancer [14]. HPVs are a circular, double-stranded DNA virus with a viral genome of approximately 8000 base pairs size that encodes two regulatory proteins (E1 and E2), three oncoproteins (E5, E6, E7), and two structural capsid proteins (L1 and L2) [15]. HPV-16 is most commonly found in OPC [6, 7]. Malignant transformation and maintenance of phenotype in head and neck cancer has been attributed mainly to E6 and E7 oncoproteins as described in cervical carcinoma [16, 17]. Experimental data shows that silencing the expression of E6 and E7 oncogenes in HPV16-positive human oropharyngeal squamous cell lines resulted in activation of the p53 and Rb tumor suppressor pathways and induction of apoptosis indicating that the two oncoproteins are needed for maintaining malignant phenotype and proliferation [18].

E6 is coded at the 5′ early viral genome and is well conserved among viruses. E6 viral transcript can be spliced leading to two spliced versions of E6, namely, E6*I and E6*II mRNA. Unspliced E6 transcript gives rise to a 19 kDa protein that forms a complex with a ubiquitin protein ligase (E6AP) that will lead to ubiquitination of p53 tumor suppressor protein and its subsequent degradation [19, 20]. The functions of p53 include regulation of cell cycle by controlling the G1 transition to the S phase at checkpoint by inducing expression of cyclin inhibitors p16, p21, and p27 [21]. Therefore, E6 oncoprotein deregulates both G1/S and G2/M cell cycle checkpoints upon DNA damage and other cellular stress leading to genomic instability. Spliced E6*I and E6*II give rise to nearly identical 6 kDa proteins. Full length E6 and E6*I can both cooperate with E7 and* ras* to transform cells* in vitro* [22]. E6 oncoprotein has also the ability to activate cellular telomerase through the transcriptional upregulation of the rate-limiting catalytic subunit of human telomerase hTERT [23]. Maintenance of telomere length has been recognized as an important step in cellular immortalization and transformation [24].

High-risk HPV E7 oncoproteins have the ability to initiate DNA synthesis in differentiated epithelial cells mainly by binding and inactivating the Rb apoptosis/tumor suppressor gene and its associated pocket proteins p107 and p130 [25]. Inactivation of Rb family of proteins by E7 results in overexpression of E2F transcription factor with upregulation of cell cycle genes resulting in the transition of cell from G1 to S phase and an increase in cell proliferation [26]. Inactivation of pRb results in increased levels of p16/CDKN2A, an inhibitor of cdk4/cyclin D and cdk6/cyclin D due to feedback loop control mechanisms [27]. Therefore, high level of p16/CDKN2A expression serves as a specific diagnostic biomarker for tumor infected with high-risk HPV [28]. E7 oncoproteins are also able to associate with either histone acetyl transferases (HATs) or histone deacetylases (HDACs) thereby influencing histone acetylation in regulatory regions for gene transcription [29]. Furthermore, several studies indicate that E6 and E7 have multiple binding partners that exert oncogenic effects beyond degradation of p53 and pRb and have complementary effects.

## 3. HPV/p16-Positive Tumors and Increased Radiosensitivity: Intrinsic Pathway

Many clinical studies have shown that patients with HPV/p16-positive tumors exhibit a far better prognosis compared to HPV/p16-negative ones when treated by primary radiochemotherapy (RCT) or RCT after surgery [30]. Despite these large clinical data confirming that HPV/p16 positivity is a prognostic marker, to date only few clinical trials are designed to use HPV/p16 positivity as a predictive marker with settings that involve treatment deescalation (e.g., RTOG 1016, DeESCALaTE HPV, and ECOG 1308). One reason might be that biological evidence is still needed for a better understanding of potential benefits of treatment deescalation.

Radiosensitivity is mainly due to ability of the cell to sense DNA damage and to control its repair, though tumor microenvironment (e.g., the oxygenation status) is also determinant for response to radiotherapy [31]. The most deleterious radiation-induced damages are double strand breaks (DSBs) and among early signals of cellular response to DSB there is phosphorylation of protein histone H2AX [32]. Unrepaired DSBs might lead to mitotic catastrophe or apoptosis, which are mechanisms partially controlled by p53 [33]. To date, there are very few experimental evidences of increased radiosensitivity in HPV/p16-positive HNSCC. In a recent paper, Rieckman and colleagues studied radiation response of 5 HPV/p16-positive HNSCC cell lines versus 5 HPV/p16-negative ones and demonstrated, on average, decreased survival fraction of HPV/p16-positive cells after irradiation. They also described increased levels of DSB and extensive G2 arrest indicating compromised DNA repair capacity in HPV/p16-positive cell lines [34]. Similar results were obtained by Kimple et al. where in addition they used a genome-wide microarray to compare gene expression between HPV/p16-positive cell lines and HPV/p16-negative cell lines, 24 h following irradiation. Results indicated multiple genes in TP53 pathway upregulated in HPV/p16-positive cells. In this same study, increased levels of apoptosis in HPV/p16-positive cell lines after irradiation could be abrogated by knockdown of TP53 through siRNA. The authors conclude that low levels of functioning p53 in HPV/p16-positive cells could be activated by radiotherapy, leading to cell death and providing evidence for enhanced radiosensitivity in HPV/p16-positive cells [35]. However, the role of enhanced apoptosis in radiosensitivity is still subject to controversy. Indeed, it has been reported that large variations in apoptosis do not lead to any changes in eventual cell killing [36–38] or that the status of p53 does not affect sensitivity to DNA-damaging agents [39]. One of the explanations for these discrepancies relies on the fact that cells do not die immediately after radiotherapy and this is highly dependent upon the cell type being investigated [40].

Despite the two previously cited papers demonstrating increased radiosensitivity of HPV/p16-positive cells [34, 35], there is no clear evidence for the implication of viral oncoproteins in radioresponse, and data published so far are inconclusive or even sometimes conflicting. For example, investigation of cell cycle and surviving fraction after low dose rate irradiation (0,025 Gy/h) in p53 wt human colon carcinoma cells engineered to express E6 and E7 showed increased levels of p53 and p21 and enhanced cell cycle arrest at G1 and G2 but no difference in clonogenic survival [41]. A study on cervix carcinoma cell lines showed no intrinsic radiosensitivity when E6 and E7 were knocked down [42]. Another publication reports that the HPV-negative C33 cervix carcinoma cell line shifts to a more radioresistant phenotype when HPV16 E6 is overexpressed [43]. The discrepancy among these results might be explained in part by differences in the type of cells studied and their different genetic background. In a more recent publication, stable expression of specific splicing-derived E6*I, E6*II, or E6 in oropharyngeal SCC showed radiosensitivity for cells expressing E6*I and total E6 supporting a link with p53 pathway [44].

Finally, there are few studies indicating that HPV oncoproteins might compromise DNA damage repair mechanisms, a strategy used by viruses to facilitate viral genome integration in the host. In HPV type 1, 8 but also 16, E6 oncoprotein has been shown to bind to XRCC1, a protein required for the repair of DNA single strand breaks and genetic stability [45]. In addition, E6 has also been shown to impair the fidelity of DNA end-joining [46].

In summary, although increased radiosensitivity of HPV/p16-positive HNSCC has been partially confirmed experimentally, further studies are still needed to identify molecular actors implicated in radioresponse of HPV/p16-positive HNSCC.

## 4. HPV/p-16-Positive Tumors and Increased Radiosensitivity: Tumor Microenvironment

As mentioned before, although radiosensitivity is mainly due to ability of the cell to sense DNA damage and to control its repair, oxygenation status of the tumor might also be determinant for response to radiotherapy [47].

In this context, retrospective analysis of DAHANCA-5 trial showed that HPV/p16-positive tumors have a superior outcome after fractionated radiotherapy compared to patients with HPV/p16-negative tumors. Patients in this study also received the hypoxic cell radiosensitizer nimorazole and the use of this drug during radiotherapy improved locoregional tumor control only in the HPV/p16-negative group. Surprisingly, HPV/p16-positive tumors seemed to be insensitive to the hypoxic modification and showed no benefit from treatment with nimorazole. The authors suggested that HPV/p16-positive HNSCCs are less hypoxic than negative ones and that this apparent lack of radiobiological relevant hypoxia in HPV/p16-positive tumors could contribute to the superior prognosis observed [48]. However, no significant association between HPV status in HNSCC and tumor hypoxia was detected by either pO2 measurements or immunohistochemical (IHC) staining for CAIX [49]. A study using hypoxia-gene expression profile demonstrated the same frequencies of hypoxia between HPV/p16-positive and -negative HNSCC tumors [50] and hypoxic status assessed by FAZA PET scans resulted also in no difference between HPV/p16-positive and -negative HNSCC patients [51]. In addition, recent* in vitro* data comparing radioresponse of HPV/p16-positive and -negative cell lines under hypoxia indicated no difference in terms of regulated gene patterns by hypoxia, whereas HPV/p16-positive cells displayed resistance under hypoxia with an oxygen enhancement ratio (OER) similar to HPV/p16-negative ones [52]. Therefore, it seems unlikely that improved prognosis of HPV/p16-positive HNSCC relies on differences in hypoxic fraction.

Another possibility is the implication of other tumor microenvironmental factors like components of the immune system. It is indeed suggested that HPV-positive tumors carry viral antigens that elicit T-cell responses that might participate in a tumor rejection process and carry out a long-term immunosurveillance. Along this line, most but not all HPV/p16-positive HNSCCs display a strong tumor infiltration by T cells [49]. However, there are very few reports on HPV16-specific T-cell immunity in HNSCC patients. They describe elevated levels of circulating HPV16 E7-specific CD8+ T cells [53] and HPV16-specific IFN*γ*-producing T cells in cultures of PBMC from patients with HPV16+HNSCC [54]. In addition, circulating anti-HPV16 antibodies have been detected in HNSCC patients with high viral load and the anti-HPV antibody status was suggested to correlate with clinical outcome [7, 55].

However, if T-cell immunity detects HPV-positive HNSCC, it probably occurs early during tumor development meaning that clinically detectable tumors have probably been immunoselected, a process identified decades ago [56, 57] and recently renamed “cancer immunoediting” [58]. Immune-mediated tumor rejection requires the activation of antitumor T cells, their differentiation into cytolytic T cells (CTL) that can reach the tumor site(s), recognition of their target antigens, and activation of effector functions. These processes have been studied in detail in HPV-induced cervical cancers, and many groups have contributed to the conclusion that several different strategies of immune evasion are used by HPV-induced (pre-)malignancies [59]. Indeed, it has been described that in addition to intracellular immune evasion mechanisms, HPVs are able to modulate host immune response through polarization of T cell subtypes, inhibition of the CTL response, and modulation of antigen presenting cells (APC) trafficking [59]. As a result, it has been shown that CD4+ and CD8+ type 1 cytokine producing T cells, reactive to E6- and E7-encoded antigens, have a positive impact on disease outcome but are weak or even undetectable in patients with progressive disease [60].

Most ongoing research is dedicated to new approaches for enhancing antitumor immune response either by increasing T-cell responses directly or by reducing various immunosuppressive mechanisms at work in the tumor microenvironment. However very little is known on the immunogenic potential of radiotherapy [61]. Irradiation-dependent tumor cell apoptosis is a potential source of tumor antigens, but whether or not it provides the appropriate inflammatory signals leading to immunogenicity of these antigens remains controversial [62]. It has been shown* in vitro* that radiotherapy induces calreticulin membrane exposure in some cancer cell lines [63]. The combination of cisplatin and radiation, commonly used to treat cervical cancer, induces calreticulin exposure, HMBG1 and ATP release, and three signals that accompany the process of “immunogenic cell death.” Interestingly, calreticulin exposure has been described as an ancestral stress response that is able to be subverted by viruses, including HPVs [64, 65].

It is then possible that radio-/chemotherapy primes the immune system to target HPV positive cancer cells. In mouse models of HPV-induced cancers, cisplatin followed by vaccination resulted in stronger HPV-specific T-cell responses, increased T-cell infiltration of the tumor [66], and increased sensitivity to CTL killing, probably through a cisplatin-induced upregulation of MHC class I in the tumor cells [67]. Remarkably, similar results were obtained with vaccination and radiation instead of chemotherapy [68, 69]. Indeed, in an* in vivo* mouse model HPV-positive tumors were more sensitive to radiation and exhibited complete clearance at 20 Gy, compared to HPV-negative counterparts, which showed persistent growth. However, radiation therapy in immune-incompetent mice was enabled to cure tumors. Adoptive transfer of wild-type immune cells into immune-incompetent mice restored HPV-positive tumor clearance with cisplatin therapy [69]. In addition, the same team has recently shown that radiation induces loss of cell surface CD47 in HPV-positive tumors [70]. CD47 is a transmembrane protein and a marker of self and its binding to antigen-presenting cells enforces tolerance [71].

## 5. Conclusion

Although there is a growing amount of data supporting the hypothesis that HPV-related tumors have a better survival due to a higher sensitivity to chemo-/radiation therapy, it is difficult to conclude that the improved clinical outcome of HPV-related HNSCC is only attributable to the intrinsic radiosensitivity of the HPV-infected cells. More likely is a complex interaction among intrinsic mechanisms of radioresponse and the tumor microenvironment including cells of the immune system.

In conclusion, HPV+ HNSCC is a distinct clinical entity with a favorable prognosis. These tumors respond better to radiotherapy even though there is little evidence for increased radiosensitivity. In other models radiotherapy cooperates with antitumor immunity, providing a rationale to investigate immune responses in HPV+ tumors after radiotherapy.

## References

[1] Gillison ML, Koch WM, Capone RB (2000). Evidence for a causal association between human papillomavirus and a subset of head and neck cancers. *Journal of the National Cancer Institute*.

[2] Andl T, Kahn T, Pfuhl A (1998). Etiological involvement of oncogenic human papillomavirus in tonsillar squamous cell carcinomas lacking retinoblastoma cell cycle control. *Cancer Research*.

[3] Argiris A, Karamouzis MV, Raben D, Ferris RL (2008). Head and neck cancer. *The Lancet*.

[4] D’Souza G, Kreimer AR, Viscidi R (2007). Case-control study of human papillomavirus and oropharyngeal cancer. *The New England Journal of Medicine*.

[5] Gillison ML, Alemany L, Snijders PJF (2012). Human papillomavirus and diseases of the upper airway: head and neck cancer and respiratory papillomatosis. *Vaccine*.

[6] Muñoz N, Bosch FX, de Sanjosé S (2003). Epidemiologic classification of human papillomavirus types associated with cervical cancer. *The New England Journal of Medicine*.

[7] Kreimer AR, Clifford GM, Boyle P, Franceschi S (2005). Human papillomavirus types in head and neck squamous cell carcinomas worldwide: a systemic review. *Cancer Epidemiology Biomarkers and Prevention*.

[8] Lassen P, Eriksen JG, Hamilton-Dutoit S, Tramm T, Alsner J, Overgaard J (2009). Effect of HPV-associated p16INK4A expression on response to radiotherapy and survival in squamous cell carcinoma of the head and neck. *Journal of Clinical Oncology*.

[9] Fakhry C, Westra WH, Li S (2008). Improved survival of patients with human papillomavirus-positive head and neck squamous cell carcinoma in a prospective clinical trial. *Journal of the National Cancer Institute*.

[10] Licitra L, Perrone F, Bossi P (2006). High-risk human papillomavirus affects prognosis in patients with surgically treated oropharyngeal squamous cell carcinoma. *Journal of Clinical Oncology*.

[11] Weinberger PM, Yu Z, Haffty BG (2004). Prognostic significance of p16 protein levels in oropharyngeal squamous cell cancer. *Clinical Cancer Research*.

[12] Petrelli F, Sarti E, Barni S (2013). Predictive value of human papillomavirus in oropharyngeal carcinoma treated with radiotherapy: an updated systematic review and meta-analysis of 30 trials. *Head and Neck*.

[13] Braakhuis BJM, Snijders PJF, Keune W-JH (2004). Genetic patterns in head and neck cancers that contain or lack transcriptionally active human papillomavirus. *Journal of the National Cancer Institute*.

[14] Doorbar J, Quint W, Banks L (2012). The biology and life-cycle of human papillomaviruses. *Vaccine*.

[15] McLaughlin-Drubin ME, Münger K (2009). Oncogenic activities of human papillomaviruses. *Virus Research*.

[16] Jabbar S, Strati K, Shin MK, Pitot HC, Lambert PF (2010). Human papillomavirus type 16 E6 and E7 oncoproteins act synergistically to cause head and neck cancer in mice. *Virology*.

[17] Jastreboff AM, Cymet T (2002). Role of the human papilloma virus in the development of cervical intraepithelial neoplasia and malignancy. *Postgraduate Medical Journal*.

[18] Rampias T, Sasaki C, Weinberger P, Psyrri A (2009). E6 and E7 gene silencing and transformed phenotype of human papillomavirus 16-positive oropharyngeal cancer cells. *Journal of the National Cancer Institute*.

[19] Scheffner M, Werness BA, Huibregtse JM, Levine AJ, Howley PM (1990). The E6 oncoprotein encoded by human papillomavirus types 16 and 18 promotes the degradation of p53. *Cell*.

[20] Bernard X, Robinson P, Nominé Y (2011). Proteasomal degradation of p53 by human papillomavirus E6 oncoprotein relies on the structural integrity of p53 core domain. *PLoS ONE*.

[21] Kessis TD, Slebos RJ, Nelson WG (1993). Human papillomavirus 16 E6 expression disrupts the p53-mediated cellular response to DNA damage. *Proceedings of the National Academy of Sciences of the United States of America*.

[22] Yamada T, Yamashita T, Nishikawa T, Fujimoto S, Fujinaga K (1995). Biologic activity of human papillomavirus type 16 E6/E7 cDNA clones isolated from SiHa cervical carcinoma cell line. *Virus Genes*.

[23] James MA, Lee JH, Klingelhutz AJ (2006). HPV16-E6 associated hTERT promoter acetylation is E6AP dependent, increased in later passage cells and enhanced by loss of p300. *International Journal of Cancer*.

[24] Kiyono T, Foster SA, Koop JI, McDougall JK, Galloway DA, Klingelhutz AJ (1998). Both Rb/p16(INK4a) inactivation and telomerase activity are required to immortalize human epithelial cells. *Nature*.

[25] Doorbar J (2005). The papillomavirus life cycle. *Journal of Clinical Virology*.

[26] Strati K, Lambert PF (2007). Role of Rb-dependent and Rb-independent functions of papillomavirus E7 oncogene in head and neck cancer. *Cancer Research*.

[27] Khleif SN, Degregori J, Yee CL (1996). Inhibition of cyclin D-CDK4/CDK6 activity is associated with an E2F-mediated induction of cyclin kinase inhibitor activity. *Proceedings of the National Academy of Sciences of the United States of America*.

[28] Klaes R, Friedrich T, Spitkovsky D (2001). Overexpression of p16ink4a as a specific marker for dysplastic and neoplastic epithelial cells of the cervix uteri. *International Journal of Cancer*.

[29] Brehm A, Nielsen SJ, Miska EA (1999). The E7 oncoprotein associates with Mi2 and histone deacetylase activity to promote cell growth. *EMBO Journal*.

[30] Ang KK, Sturgis EM (2012). Human papillomavirus as a marker of the natural history and response to therapy of head and neck squamous cell carcinoma. *Seminars in Radiation Oncology*.

[31] Martinive P, Defresne F, Bouzin C (2006). Preconditioning of the tumor vasculature and tumor cells by intermittent hypoxia: implications for anticancer therapies. *Cancer Research*.

[32] Kinner A, Wu W, Staudt C, Iliakis G (2008). Gamma-H2AX in recognition and signaling of DNA double-strand breaks in the context of chromatin. *Nucleic Acids Research*.

[33] Eriksson D, Stigbrand T (2010). Radiation-induced cell death mechanisms. *Tumor Biology*.

[34] Rieckmann T, Tribius S, Grob TJ (2013). HNSCC cell lines positive for HPV and p16 possess higher cellular radiosensitivity due to an impaired DSB repair capacity. *Radiotherapy and Oncology*.

[35] Kimple RJ, Smith MA, Blitzer GC (2013). Enhanced radiation sensitivity in HPV-positive head and neck cancer. *Cancer Research*.

[36] Aldridge DR, Arends MJ, Radford IR (1995). Increasing the susceptibility of the rat 208F fibroblast cell line to radiation-induced apoptosis does not alter its clonogenic survival dose-response. *The British Journal of Cancer*.

[37] Kyprianou N, King ED, Bradbury D, Rhee JG (1997). bcl-2 over-expression delays radiation-induced apoptosis without affecting the clonogenic survival of human prostate cancer cells. *International Journal of Cancer*.

[38] Wouters BG, Giaccia AJ, Denko NC, Brown JM (1997). Loss of p21(Waf1/Cip1) sensitizes tumors to radiation by an apoptosis-independent mechanism. *Cancer Research*.

[39] Wouters BG, Denko NC, Giaccia AJ, Brown JM (1999). A p53 and apoptotic independent role for p21(waf1) in tumour response to radiation therapy. *Oncogene*.

[40] Dewey WC, Ling CC, Meyn RE (1995). Radiation-induced apoptosis: relevance to radiotherapy. *International Journal of Radiation Oncology Biology Physics*.

[41] Deweese TL, Walsh JC, Dillehay LE (1997). Human papillomavirus E6 and E7 oncoproteins alter cell cycle progression but not radiosensitivity of carcinoma cells treated with low-dose-rate radiation. *International Journal of Radiation Oncology Biology Physics*.

[42] Kamradt MC, Mohideen N, Krueger E, Walter S, Vaughan ATM (2000). Inhibition of radiation-induced apoptosis lay dexamethasone in cervical carcinoma cell lines depends upon increased HPV E6/E7. *The British Journal of Cancer*.

[43] Hampson L, El Hady ES, Moore JV, Kitchener H, Hampson IN (2001). The HPV16 E6 and E7 proteins and the radiation resistance of cervical carcinoma. *The FASEB Journal*.

[44] Pang E, Delic NC, Hong A, Zhang M, Rose BR, Lyons JG (2011). Radiosensitization of oropharyngeal squamous cell carcinoma cells by human papillomavirus 16 oncoprotein E6I. *International Journal of Radiation Oncology Biology Physics*.

[45] Iftner T, Elbel M, Schopp B (2002). Interference of papillomavirus E6 protein with single-strand break repair by interaction with XRCC1. *EMBO Journal*.

[46] Shin K-H, Ahn JH, Kang MK (2006). HPV-16 E6 oncoprotein impairs the fidelity of DNA end-joining via p53-dependent and -independent pathways. *International Journal of Oncology*.

[47] Yoshimura M, Itasaka S, Harada H, Hiraoka M (2013). Microenvironment and radiation therapy. *BioMed Research International*.

[48] Lassen P, Eriksen JG, Hamilton-Dutoit S (2010). HPV-associated p16-expression and response to hypoxic modification of radiotherapy in head and neck cancer. *Radiotherapy and Oncology*.

[49] Kong CS, Narasimhan B, Cao H (2009). The relationship between human papillomavirus status and other molecular prognostic markers in head and neck squamous cell carcinomas. *International Journal of Radiation Oncology Biology Physics*.

[50] Toustrup K, Sørensen BS, Lassen P (2012). Gene expression classifier predicts for hypoxic modification of radiotherapy with nimorazole in squamous cell carcinomas of the head and neck. *Radiotherapy and Oncology*.

[51] Mortensen LS, Johansen J, Kallehauge J (2012). FAZA PET/CT hypoxia imaging in patients with squamous cell carcinoma of the head and neck treated with radiotherapy: results from the DAHANCA 24 trial. *Radiotherapy and Oncology*.

[52] Sørensen BS, Busk M, Olthof N (2013). Radiosensitivity and effect of hypoxia in HPV positive head and neck cancer cells. *Radiotherapy and Oncology*.

[53] Albers A, Abe K, Hunt J (2005). Antitumor activity of human papillomavirus type 16 E7-specific T cells against virally infected squamous cell carcinoma of the head and neck. *Cancer Research*.

[54] Hoffmann TK, Arsov C, Schirlau K (2006). T cells specific for HPV16 E7 epitopes in patients with squamous cell carcinoma of the oropharynx. *International Journal of Cancer*.

[55] Smith EM, Pawlita M, Rubenstein LM, Haugen TH, Hamsikova E, Turek LP (2010). Risk factors and survival by HPV-16 E6 and E7 antibody status in human papillomavirus positive head and neck cancer. *International Journal of Cancer*.

[56] Uyttenhove C, Maryanski J, Boon T (1983). Escape of mouse mastocytoma P815 after nearly complete rejection is due to antigen-loss variants rather than immunosuppression. *Journal of Experimental Medicine*.

[57] Maryanski JL, Marchand M, Uyttenhove C, Boon T (1983). Immunogenic variants obtained by mutagenesis of mouse mastocytoma P815. VI. Occasional escape from host rejection due to antigen-loss secondary variants. *International Journal of Cancer*.

[58] Dunn GP, Bruce AT, Ikeda H, Old LJ, Schreiber RD (2002). Cancer immunoediting: from immunosurveillance to tumor escape. *Nature Immunology*.

[59] Grabowska AK, Riemer AB (2012). The invisible enemy—how human papillomaviruses avoid recognition and clearance by the host immune system. *The Open Virology Journal*.

[60] Piersma SJ, Jordanova ES, van Poelgeest MIE (2007). High number of intraepithelial CD8+ tumor-infiltrating lymphocytes is associated with the absence of lymph node metastases in patients with large early-stage cervical cancer. *Cancer Research*.

[61] Durante M, Reppingen N, Held KD (2013). Immunologically augmented cancer treatment using modern radiotherapy. *Trends in Molecular Medicine*.

[62] McBride WH, Chiang C-S, Olson JL (2004). A sense of danger from radiation. *Radiation Research*.

[63] Obeid M, Panaretakis T, Joza N (2007). Calreticulin exposure is required for the immunogenicity of *γ*-irradiation and UVC light-induced apoptosis. *Cell Death and Differentiation*.

[64] Kepp O, Senovilla L, Galluzzi L (2009). Viral subversion of immunogenic cell death. *Cell Cycle*.

[65] Regan JA, Laimins LA (2008). Bap31 is a novel target of the human papillomavirus E5 protein. *Journal of Virology*.

[66] Tseng CW, Monie A, Trimble C (2008). Combination of treatment with death receptor 5-specific antibody with therapeutic HPV DNA vaccination generates enhanced therapeutic anti-tumor effects. *Vaccine*.

[67] Sin J-I, Kim J-M, Bae SH, Lee I, Park JS, Ryoo HM (2009). Adoptive transfer of human papillomavirus E7-specific CTL enhances tumor chemoresponse through the perforin/granzyme-mediated pathway. *Molecular Therapy*.

[68] Tseng C-W, Trimble C, Zeng Q (2009). Low-dose radiation enhances therapeutic HPV DNA vaccination in tumor-bearing hosts. *Cancer Immunology, Immunotherapy*.

[69] Spanos WC, Nowicki P, Lee DW (2009). Immune response during therapy with cisplatin or radiation for human papillomavirus-related head and neck cancer. *Archives of Otolaryngology—Head and Neck Surgery*.

[70] Vermeer DW, Spanos WC, Vermeer PD, Bruns AM, Lee KM, Lee JH (2013). Radiation-induced loss of cell surface CD47 enhances immune-mediated clearance of human papillomavirus-positive cancer. *International Journal of Cancer*.

[71] Sarfati M, Fortin G, Raymond M, Susin S (2008). CD47 in the immune response: role of thrombospondin and SIRP-alpha reverse signaling. *Current Drug Targets*.

